# In Vitro 3D Models of Haematological Malignancies: Current Trends and the Road Ahead?

**DOI:** 10.3390/cells14010038

**Published:** 2025-01-02

**Authors:** Carlotta Mattioda, Claudia Voena, Gianluca Ciardelli, Clara Mattu

**Affiliations:** 1 DIMEAS, Politecnico di Torino, C.so Duca degli Abruzzi 24, 10129 Torino, Italy; carlotta.mattioda@polito.it (C.M.); gianluca.ciardelli@polito.it (G.C.); 2 Department of Molecular Biotechnology and Health Sciences, Molecular Biotechnology Center, University of Torino, 10126 Torino, Italy; claudia.voena@unito.it

**Keywords:** in vitro models, haematological malignancies, tumor microenvironment

## Abstract

Haematological malignancies comprise a diverse group of life-threatening systemic diseases, including leukaemia, lymphoma, and multiple myeloma. Currently available therapies, including chemotherapy, immunotherapy, and CAR-T cells, are often associated with important side effects and with the development of drug resistance and, consequently, disease relapse. In the last decades, it was largely demonstrated that the tumor microenvironment significantly affects cancer cell proliferation and tumor response to treatment. The development of biomimetic, in vitro models may promote the investigation of the interactions between cancer cells and the tumor microenvironment and may help to better understand the mechanisms leading to drug resistance. Although advanced in vitro models have been largely explored in the field of solid tumors, due to the complex nature of the blood cancer tumor microenvironment, the mimicking of haematological malignancies mostly relies on simpler systems, often limited to two-dimensional cell culture, which intrinsically excludes the microenvironmental niche, or to ethically debated animal models. This review aims at reporting an updated overview of state-of-the-art hematological malignancies 3D in vitro models, emphasizing the key features and limitations of existing systems to inspire further research in this underexplored field.

## 1. Introduction

Haematological malignancies are life-threatening, systemic diseases, commonly referred to as blood cancers (BCs), and they were responsible for nearly 0.7 million deaths worldwide in 2020 [1,2]. BCs involve a diverse range of neoplasms arising from cells within the bone marrow (BM) and the lymphatic system and are categorized into myeloid and lymphoid tumors, depending on the altered precursor cells. BCs include three main classes of malignancies: leukaemia, myeloma, and lymphoma [3].

Leukaemia is characterized by the malignant clonal expansion of sub-populations of hematopoietic stem cells (HSPCs) within the BM and is further categorized according to the mutated precursor cell type (i.e., lymphoid or myeloid) and disease progression pace (i.e., acute or chronic) [4]. Thus, leukaemia encompasses the following conditions: acute lymphocytic leukaemia (ALL), which arises from mutated lymphoblasts and is commonly diagnosed at paediatric age; chronic lymphocytic leukaemia (CLL), which is a consequence of B-lymphocytes alteration, and acute or chronic myeloid leukaemia (AML or CML) [5], which arise, respectively, from myeloid progenitors and myeloblasts. 

Multiple myeloma (MM) is a B-cell malignancy which affects completely differentiated plasma cells in the BM [6,7,8,9]. During B-lymphocyte development, genetic alterations may lead to the development of MM cells [10]. MM cells progressively reduce the available space for normal cells in the BM, induce osteoclasts recruitment, and interfere with MSCs differentiation into osteoblasts, causing abnormal bone resorption [11,12,13].

The third category of BCs consists of malignant lymphomas, which originate from the clonal expansion of lymphocytes [14,15]. Lymphomas are classified into Hodgkin lymphoma (HL) and non-Hodgkin’s lymphomas (NHLs), which include mature B-cell-, mature T-cell-, and natural killer (NK)-cell neoplasms. 

HL represents one of the most common tumors in young adults. Primary HL has a promising prognosis, with a 5-year survival rate around 98% [16], while the long-term survival rate remains poor due to relapsed or refractory disease [17]. Histologically, HL is classified in classical HL (cHL) and nodular lymphocyte-predominant HL. Classical HL accounts for almost 95% of all diagnosed HLs and is characterized by the presence of CD30+ Reed–Sternberg [18], large multinucleated cells derived from B-lymphocytes, embedded in a tumor microenvironment (TME) rich in immune cells [19,20,21], while nodular lymphocyte predominant HL is characterized by the presence of CD20+ lymphocyte-predominant (LP) cells, surrounded by a TME mostly composed of mature lymphocytes [22].

NHLs include a vast group of BCs, which commonly develop in the lymph nodes but which can occur in almost any lymphoid tissue [23], arising from B-lymphocytes (i.e., follicular lymphoma, Burkitt lymphoma, diffuse large B-cell lymphoma, mantle cell lymphoma, marginal zone lymphoma, and primary central nervous system lymphoma), or from T-lymphocytes or NK-cells (i.e., adult T-cell lymphoma and mycosis fungoides) [24]. In a physiological context, lymphoid progenitors, presenting both B- and T-cell potential, originate in the BM followed by migration of T-cell progenitors to the thymus, and of B-lymphocytes to secondary lymphoid organs (SLOs) (e.g., lymph nodes and spleen). Maturation arrest during lymphocyte development or the establishment of proliferative and anti-apoptotic alterations may lead to a lymphoid neoplasm [25]. The mutated lymphocytes proliferate in an abnormal way and accumulate within specific sections of the lymphatic system, losing their infection-fighting ability [26].

Multiple therapeutic options are available for the management of BCs, including radiotherapy, chemotherapy, targeted therapy, immunotherapy, and chimeric antigen receptor (CAR)-T cell therapy (as summarized in Table 1). Nowadays, treatment consists of combinations of multiple agents, including chemotherapy, proteasome inhibition, immunomodulation, and/or monoclonal antibodies [27]. Unfortunately, only in a small subset of cases can long-term remission be successfully achieved. Traditional treatments based on radiotherapy and/or chemotherapy present systemic toxicity and off-target effects [28,29]. Moreover, the development of drug resistance resulting in treatment-refractory disease is common and represents the leading cause of mortality in BC patients [30]. The TME in BCs has been suggested to play a fundamental role in driving drug resistance and recurrence, albeit the molecular mechanisms governing this process still need to be elucidated [31]. To date, in vitro pre-clinical research on BCs mostly relied on 2D cultures, which intrinsically exclude the microenvironmental niche [32], resulting in the underestimation of the TME role. Three-dimensional in vitro cancer models have the potential to reproduce the TME and its complex interactions with cancer cells. Unfortunately, their application has remained mostly confined to solid tumors [33,34,35], with only a few applications in BCs. Reliable in vitro models mimicking BCs may facilitate the investigation of the tumor/TME interactions and the understanding of the mechanisms governing relapse and drug resistance, and may support the design of more efficient therapies. Unfortunately, the complex nature of the stromal environment in lymph nodes, BM, and SLOs, characterized by a broad variety of cell populations in different differentiation stages [36,37], as well as the characteristics of the tumor cell populations (e.g., suspension growth), have limited the development of 3D models of BCs [32], with only a few attempts reported in the literature.

In this scenario, this review aims at providing an updated overview of the current landscape of in vitro models of haematological tumors, highlighting the main desired features and constraints, with the aim of encouraging new research in this largely unexplored field.

## 2. The Tumor Microenvironment in BC 

The complex interactions between stromal cells and HPSCs govern different physiological and pathological mechanisms, such as haematopoiesis, cell survival, and cell function. These interactions occur in unique microenvironments which are characteristic of the tissue and, in BCs, of the specific disease subtype. Thus, the TME can be considered a therapeutic target due to its influence on malignant evolution and drug resistance [79]. For instance, HSPCs reside in the BM microenvironment, commonly referred to as “niches”, composed by different cell populations, including mesenchymal stem cells (MSCs), endothelial cells, sympathetic nerves, and non-myelinating Schwann cells [80]. In BCs, cancer cells and cancer-associated stem cells alter this complex microenvironment, hindering physiological haematopoiesis [81]. Therefore, understanding the composition and key features of each pathological niche is fundamental for the design of reliable in vitro models of BCs and to identify the key aspects governing malignancy, treatment response, and relapse.

### 2.1. The Bone Marrow Microenvironment 

**Figure 1:**
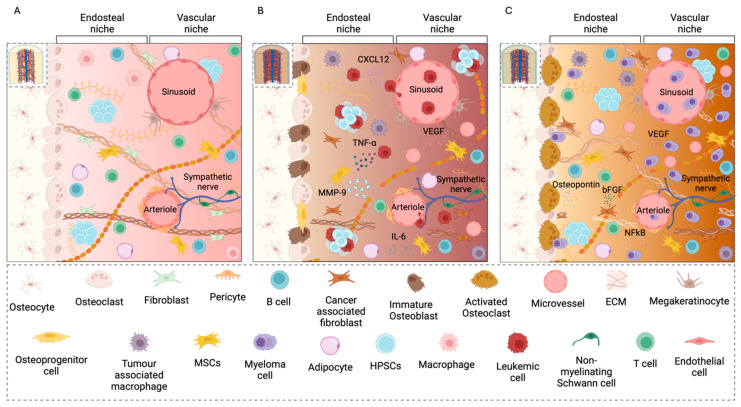
Schematic representation of BM in physiological condition (**A**) and affected by leukaemia (**B**), and multiple myeloma BM (**C**) microenvironment. [Created in BioRender. Mattu, C. https://BioRender.com/l65m769 (accessed on 19 December 2024)].

The BM is a soft tissue that fills the cavities within bones and consists of different cell types, such as immune cells, osteoblasts, osteoclasts, osteocytes, MSCs, fibroblasts, stromal cells, and vascular endothelial cells (Figure 1A) [82]. The ECM is well-vascularized and is mainly composed of fibronectin, collagen I, II, III, IV, and X, laminin, tenascin, thrombospondin, and elastin [83]. In physiological conditions, the BM microenvironment is known to influence HPSCs’ fate, for instance, through interactions with the stromal cell population [84,85,86,87]. Two main niches can be identified in the BM: the endosteal, or osteoblastic, niche and the vascular, or sinusoidal, niche [88]. The endosteal niche includes osteoblasts and osteoclasts, while the vascular niche is composed by the sinusoids, a microvascular network through which the bloodstream interacts with the BM [89]. In the niches, non-hematopoietic cells interact with HPSCs through the secretion of cytokines, chemokines, and other soluble factors, governing the proliferation, differentiation, adhesion, and quiescence of HPSCs [90,91]. For example, the hypoxic microenvironment in the endosteal niche maintains the HPSCs in a quiescent state, while the vascular niche supports proliferating and differentiating progenitor cells to form the hematopoietic cell populations [92].

In BCs, the BM structure and composition are affected in different ways depending on the tumor subtype [93,94]. For instance, in leukaemia (Figure 1B) the altered balance between bone formation and resorption in the osteoblastic niche leads to bone loss [95] and to deficient bone mineralization [96,97]. The vascular niche is also altered in leukaemia. It has been shown that endothelial cells and leukemic cells interact through autocrine and paracrine stimuli, leading to the attachment of tumor cells to the endothelium [98]. This behaviour allows the migration of leukemic stem cells within the vascular network, and, consequently, the metastatic development. Deregulated angiogenesis is another characteristic aspect of the leukaemia microenvironment, with new blood vessel formation being fundamental for the progression of this pathology. Indeed, the micro vessel density in the BM has been identified as a prognostic marker in leukaemia [99]. The ECM also plays a crucial role in leukaemia development and progression [100]. In ALL, B-cells and leukemic cells secrete tumor necrosis factor α (TNF-α), leading to an increased production of matrix metalloproteinase 9 (MMP-9) by MSCs, resulting in ECM degradation, and, ultimately, in local invasion by leukemic cells [101]. In AML, the interactions between type-I Collagen and α2β1 integrins protect leukemic cells from drug-induced apoptosis, confirming the fundamental role of the ECM in driving chemotherapy resistance [102]. 

In MM, the BM microenvironment (Figure 1C) plays a crucial role in the progression of MM and in the response to therapeutic agents by facilitating immune escape and supporting tumor proliferation [103,104,105,106]. Malignant MM cells can locally modify the BM niche by altering the secretion of cytokines and exosomes, creating a growth-supporting environment which stimulates metastatic dissemination [107,108]. Typically, MM cells adhere to the endosteal niche, which maintains them in a quiescence state. Patients affected by MM often display a severe imbalance between osteoblasts and osteoclasts [109,110,111]. Indeed, remodeling of the niche by osteoclasts interrupts quiescence and causes the reactivation of MM cells and their migration away from the niche [112,113]. Interaction between MM cells and MSCs is fundamental during this process, as it triggers the release of pro-osteoclastogenic factors, such as proinflammatory cytokines (IL-6) and osteoclastogenic factors (such as RANKL) that activate osteoclasts and suppress osteoblasts [12,114,115]. Moreover, osteoclasts release osteopontin, a proangiogenic factor, which cooperates with vascular endothelial growth factor (VEGF) and basic fibroblast growth factor (bFGF), secreted by MM cells and BM stromal cells, to enhance angiogenesis in the BM microenvironment [116,117,118]. CD138 and VLA-4 expressed by MM cells facilitate their interaction with ECM proteins, such as collagen type-I and fibronectin. This interaction triggers the activation of the nuclear factor kappa-light-chain-enhancer of activated B-cells (NFkB), which induces MM cell proliferation and adhesion-mediated drug resistance [119,120]. 

In summary, the BM microenvironment in BCs is characterized by an altered equilibrium between osteoblasts and osteoclasts, an enhanced secretion of angiogenic factors and inflammatory cytokines, and an altered ECM structure and composition. These alterations play a key role in tumor growth, invasion, and in the development of drug resistance, confirming the importance of the TME in the development and progression of haematological malignancies [121].

### 2.2. The Microenvironment of Secondary Lymphoid Organs 

**Figure 2:**
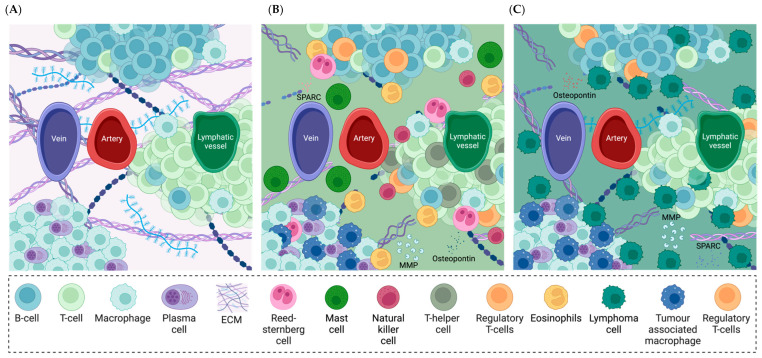
Schematic representation of lymph node microenvironment composition in physiological conditions (**A**), in Hodgkin’s lymphoma (**B**), and in non-Hodgkin’s lymphoma (**C**). [Created in BioRender. Mattu, C. https://BioRender.com/k93w241 (accessed on 19 December 2024)].

SLOs comprise lymph nodes, the spleen, Peyer’s patches, and other mucosal tissues, such as nasal-associated lymphoid tissue, adenoids, and tonsils. Each SLO is constituted by a complex network of stromal cells, which are unique to the tissue, along with blood and lymphatic endothelial structures and distinct B-cells and T-cells zones (Figure 2A) [122]. The microenvironment interacts with B- and T-cells, supporting their survival and enabling them to proceed to a different SLO, when an antigen is not identified. Conversely, when B- and T-cells recognize a specific antigen, the microenvironment in SLOs supports their activation and proliferation [37]. Peripheral lymphoid tissues are composed of two types of ECM, which differ in terms of morphology and biochemical signaling, namely the interstitial matrix and the basement membrane. The interstitial matrix supports the interaction between fibroblastic reticular cells and is mainly constituted by collagen I, III, V, and XI, proteoglycans and glycoproteins, such as fibronectin, tenascin, and vitronectin [123]. The basement membrane separates the different functional compartments of the organ, and is composed of collagen IV, laminins, heparan sulphate proteoglycans, and glycoproteins [124]. The lymphoid compartments are connected to the peripheral sites through the conduit system, which support the transport of chemokines and cytokines and allow lymphocyte locomotion inside the SLOs [125,126].

SLOs are involved in the initiation, proliferation, and spreading of HL and nHL [127,128,129]. The TME composition is extremely variable depending on the subtype of BC. For instance, in HL and in some T-cell lymphomas, tumor cells, namely Reed-Sternberg cells, represent only a small percentage of the tumor mass, while the majority is constituted by non-tumoral cells (Figure 2B). In B-cell lymphomas, the proportion of non-tumoral cells is significantly reduced (Figure 2C) [130]. Immune cells play a crucial role in lymphoma progression, by assuming either pro- or anti-oncogenic phenotypes depending on the signals from the surrounding microenvironment. Macrophages, dendritic cells, eosinophils, and tumor-infiltrated lymphocytes are generally involved in tumor control, while myeloid-derived suppressor cells, mast cells, regulatory T-cells, and tumor-associated macrophages (TAMs) are implicated in immunosuppressing mechanisms [131]. The ECM composition is also affected by tumor progression. Neoplastic and stromal cells may produce new ECM components, essential for tumor–stroma interactions and tumor proliferation [132]. For instance, SPARC (secreted protein acidic rich in cysteine) and osteopontin are non-structural ECM proteins which play a crucial role in supporting tumor growth and invasion, e.g., by regulating ECM degradation, and are often deregulated in BC [133,134,135,136].

In summary, SLOs’ microenvironment in BCs, characterized by the presence of tumor-infiltrating immune cells and by the increased secretion of non-structural proteins, is a key player in supporting cell migration and tumor growth, representing a potential therapeutic target that should not be overlooked. 

## 3. In Vitro Models of BCs

**Figure 3:**
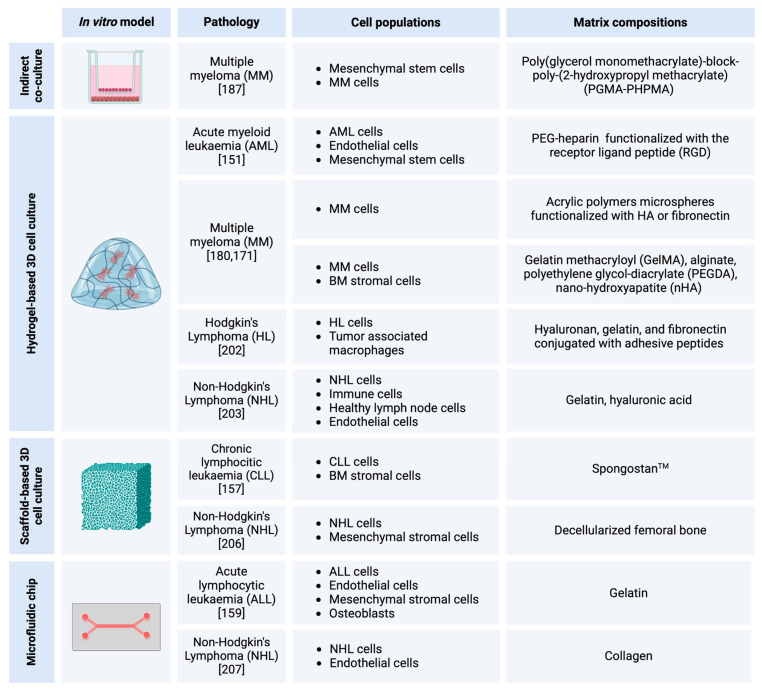
Representation of the available 3D in vitro models of hematological malignancies [Created in BioRender. Mattu, C. (2024) https://BioRender.com/m25e426 (accessed on 19 December 2024)].

As discussed above, the TME in BCs plays an active role in supporting tumor progression, metastatic dissemination, and in orchestrating treatment response. The microenvironment of both BM and SLOs, presents pathology-specific alterations which lead to variations in the ECM structure and composition, enhanced secretion of angiogenic factors and inflammatory cytokines, and migration and proliferation of stromal cells and immune cells. These alterations facilitate tumor growth, invasion, and drug resistance, highlighting the importance of the TME in the development and progression of haematological malignancies [121,137]. Therefore, the complex interactions between cancer cells and other cellular and non-cellular components of the TME should be considered to fully unravel the mechanisms underlying BC development and to design new therapeutic options. Unfortunately, in vitro studies are still mostly based on conventional 2D cell cultures, which fall short of replicating the crucial dynamics, architectural features, and the composition of the TME [94,138]. Models based on spheroids and organoids are widely used to study haematological malignancies [139,140], as they allow for the reproduction of connections among cells in a tridimensional environment in an easy and reproducible fashion. [141] However, organoid-based models are costly and often require a long maturation time. Moreover, the absence of immune and stromal components and blood vessels limits their ability to faithfully replicate the microenvironment in BCs [142]. More complex 3D systems, presented in Figure 3, have the potential to more faithfully mimic cell/TME interactions and to replicate cell colonization and proliferation within niches, therefore facilitating the identification and evaluation of potential therapeutic targets and enhancing the understanding of BC pathophysiology and recurrence [137,143]. The development of representative 3D in vitro models of BCs is extremely challenging, since different aspects, such as chemical and biochemical stimuli (e.g., calcium concentration, oxygen concentration, growth factors release), heterogeneous cell populations, topographic and mechanical proprieties of the niches, and the presence of the vascular network, should be considered simultaneously [36]. Moreover, the ex vivo culture of HPSC strictly depends on the physical and biochemical properties of the substrate in which the cells are grown, and on the presence of other cell populations in co-culture, as well as on environmental stimuli, such as hypoxia [144].

The following paragraphs report examples of the recent 3D in vitro models of different BC subtypes, highlighting their advancements over traditional 2D cultures and discussing their limitations. 

### 3.1. Leukaemia In Vitro Models 

Challenges in the development of in vitro models of leukaemia include the presence of different niches and the heterogeneity of the cell populations composing the niches [145], the presence of leukaemia-associated stem cells [146], and the complex interactions between leukaemia cells and other cell types, including endothelial cells [147], MSCs [148], and immune cells [149], and between leukaemia cells and the ECM [150]. For example, in acute leukaemias, the crosstalk between tumor cells and the vascular endothelium actively contributes to pathology progression and to the development of drug resistance [151], while MSCs are known to orchestrate the interactions between cancer cells and other cells in the TME, controlling the behaviour of leukemic cells [152,153]. The accumulation of B-lymphocytes is another key feature of BCs. Indeed, B-cell infiltration in CLL has been associated with pathology progression and with the development of drug resistance [154,155]. An additional challenge in designing leukaemia models is represented by the different composition and morphological features of the niches, which depend on the pathology subtype. 

Few attempts have been made to replicate leukaemia in vitro. Some models exploit non-contact interactions (e.g., by using trans-well inserts) among two or more cell types [156], and others are based on the co-culture of two or more cell types inside porous scaffolds or 3D hydrogels that may include ECM proteins and exploit the self-assembly ability of the cells to obtain different compartments [151,157,158]. More recent approaches harness microfluidics [159] or 3D-printing technologies to reproduce some of the morphological features of the microenvironment, together with a controlled spatial positioning of the different cell types [160]. 

For instance, Torres-Barrera et al. [156] used a co-culture model to investigate the role of the BM endothelium in regulating the quiescence and proliferation of leukaemia-associated stem cells (LSCs) in CML. To this aim, primary human CML stem cells (Lin-CD34+) were cultured for up to 72 h with or without direct contact with normal endothelial cells. When endothelial cells were co-cultured with CML cells, either in direct or indirect contact, proliferation of CML cells was observed, with an increase in the cell number of 110%–170% as compared to the initial count. On the other hand, in control cultures without endothelial cells, a reduction in the number of CML cells was noted. Similarly, culturing CML progenitor cells in contact with endothelial cells produced an increase of nearly 20% in the cell count. The number of colony-forming cells remained constant when leukemic cells were cultured in direct contact with endothelial cells, while the number of colony-forming cells decreased by 50% in co-culture without contact and by 83% in control cultures with basal medium alone. These data suggested that direct contact with endothelial cells is essential for the maintenance of the CML progenitors and stem cells. Moreover, after 72 h of co-culture with endothelial cells, two different populations of hematopoietic cells were identified: one population adhering to the endothelial layer (15.9%) and a second population which remained in suspension (84%). Analysis of the cell phenotype indicated that among the adherent population, the percentage of stem, progenitor, and mature cells was 16%, 61%, and 22%, respectively. In the floating cells, the percentage of stem, progenitor, and mature cells was 9%, 74%, and 17%, respectively. The work suggested that direct contact co-culture with endothelial cells is essential for the regulation of haematopoiesis and that the microenvironment produced by endothelial cells may facilitate the maintenance of primitive CML cells in a quiescent state.

Svozilová and co-workers [158] proposed a biocompatible polymer hydrogel functionalized with the Arg-Gly-Asp-Ser (RGDS) peptide to co-culture CLL cells together with BM stromal cells. They observed that the scaffold culture could maintain physiological cell morphology and that BM stromal cells and CLL cell lines were metabolically active for up to 70 days in the hydrogel, while patient-derived CLL cells had a significantly lower survival pf of only 4 days. 

Using a similar hydrogel-based approach, Bray et al. [151] developed a hydrogel-based 3D system including the vascular niche, to mimic the interactions between leukemic cells and endothelial cells and understand their role in pathology progression and drug resistance in AML. In detail, they developed a triculture system of endothelial cells (HUVECs), MSCs, and different leukaemia cell lines or primary leukaemia cells from AML patients (pAML) in an RGD-modified poly (ethylene glycol) (PEG)-heparin hydrogel [161,162]. The three cell populations were seeded within the matrix, using a three-to-six-fold higher endothelial cell concentration and a ten-fold lower MSCs concentration in comparison to AML cells. Growth factors (namely, VEGF, fibroblast growth factor 2, and stromal cell-derived factor 1) were added to induce the development of an endothelial cell capillary network within the gels. Interestingly, they observed that all tested AML cell lines were mainly localized in the proximity of the HUVEC-MSCs vascular network, suggesting that the co-culture system may replicate the supportive role played by the endothelial network in mediating adhesion and proliferation of AML cells. This confinement was confirmed also with primary AML cells, albeit with a lower proliferation rate. The effect of this dynamic interactions between AML cells and the vascular network on drug resistance was investigated after treatment with Daunorubicin. As expected, drug cytotoxicity was significantly higher in 2D cultures and in 3D monocultures, in comparison to the three-culture system (with a 2.5-to-30-fold increase, depending on the cell line and the testing conditions), suggesting that complex in vitro models may better recapitulate drug response. Treatment with a CXC chemokine receptor type 4 (CXCR4) inhibitor, which has been suggested to block the CXCR4/CXCR12 axis involved in AML cells protection by stromal cells, was also tested [163,164]. To investigate this aspect, the authors administered the CXCR4 antagonist AMD3100 to the AML cells in the model. They found that administration of the CXCR4 antagonist significantly reduced contact between the endothelial cells and AML cells, both for AML cell lines and for primary AML cells, replicating the expected behaviour of the molecule.

In a different work, Ma et al. [159] designed a microfluidic leukaemia-on-a-chip model simulating the ALL niche in the BM to study the interactions between ALL cells and the BM microenvironment [165]. The model included a central venous sinus, representing the medullary cavity, and endosteal regions connected to medium reservoirs to continuously provide nutrients to the cells. The chip design, with its different compartments, allowed communication between tumor cells and TME cells, reproducing the TME role in supporting ALL cells proliferation and drug resistance. 

Two different ALL cell lines with favorable and poor treatment response (i.e., REH and SUP) were seeded within the microfluidic device in combination with endothelial cells (HUVECs), MSCs, and human osteoblasts (hFOB 1.19). In detail, ALL cells were cultured in the central area of the device, the BM niche cells (endothelial cells and MSCs) were seeded in the central ring, and the human osteoblasts in the outer ring, mimicking the endosteal region, connected with four reservoirs for medium supply. Periodic cytokine quantification indicated the increased secretion of CCL2, CCL5, interleukin (IL)-6, and IL-8 with increasing ALL cell proliferation for both cell lines, as well as incremented NF-kB signaling, which was further enhanced in co-culture with the BM niche cells. Time lapse imaging highlighted the movement of endothelial cells towards the central region, confirming their attraction towards the tumor cells. The model was also used to investigate the interactions between the chemokine CXCL12 and its receptor CXCR4, which is implicated in leukaemia progression [166]. They observed that ALL cells in co-culture with BM niche cells expressed a higher amount of CXCR4 in comparison to the same cells in single culture, confirming that the interactions between ALL and BM niche cells is implicated in CXCL12/CXCR4 signalling. Moreover, the interactions between ALL cells and hFOB seeded in the outer region of the chip was analysed to investigate the role of the endosteal niche in regulating pathology progression. In the presence of ALL cells, secretion of osteopontin by osteoblasts was reduced, suggesting an effect of leukaemia cells on the differentiation and maturation of hFOB. The authors reported a higher content of p21^+^ ALL cells in the endosteal niche in comparison to the perivascular niche. Since p21 indicates dormant ALL cells, these findings confirmed that osteoblasts are involved in maintaining quiescence of ALL cells. Different therapies used to treat ALL were also tested in the model, namely prednisone (PRE), vincristine (VCR), and nilotinib (NIL). ALL cells in single culture displayed a higher sensitivity to all the tested agents, confirming the essential influence of the microenvironment on treatment response. SUP cells, which are obtained from a drug refractory ALL, showed higher resistance in comparison to REH cells in the model, confirming that drug resistance is maintained in 3D cultures. 

To investigate the influence of the TME in the progression of CLL, Barbaglio et al. [157] proposed a scaffold-based 3D system mimicking the homing and migration phase in CLL in response to treatment administration. Specifically, the authors designed a 3D model of the BM microenvironment, using a Spongostan^TM^ scaffold, inserted into a rotating bioreactor to enable the interaction between CLL and BM stromal cells. The scaffold was first seeded with a BM stromal cell line (HS5), followed by the addition of GFP-labeled CLL cells (GFP-MEC1) under rotating conditions [167]. They observed that the amount of CLL cells within the scaffold was significantly higher when CLL cells were co-cultured with stromal cells in comparison to CLL cells in single culture, confirming the role of BM stromal cells in sustaining CLL infiltration and proliferation. They also evaluated the expression of the hematopoietic lineage cell-specific protein 1 (HS1) [168] in CLL cells cultured in the BM microenvironment model. To this aim, genetically modified CLL cells with reduced expression of HS1 were co-cultured with wild type cells with high HS1 expression in the BM microenvironment model. The authors observed that wild type cells were mostly located outside the scaffold and that most of the cells in the scaffold had low HS1 expression, suggesting a better BM-homing ability for cells of low HS1 expression. Using patient-derived CLL cells from six different patients, the authors found that HS1 expression varied depending on the tissue from which the cells were obtained, with cells derived from BM showing lower HS1 expression in comparison to cells obtained from the peripheral blood of the same patient. These results suggested that the tissue-specific microenvironment controls protein expression in CLL cells, and that the in vitro BM scaffold model was able to reproduce this behaviour. The authors also investigated the response to the BTK inhibitor (ibrutinib) treatment in the model and found that both CLL cell line and primary CLL cells migrated outside the scaffold after treatment to a much higher extent than the untreated control, confirming that drug treatment influences cell mobilization [169]. 

### 3.2. Multiple Myeloma In Vitro Models

MM predominantly develops within the BM niche, where MM cells establish close interactions with the ECM, leading to the generation of signals promoting cell survival and inhibiting apoptosis [170,171]. The BM microenvironment, composed of hematopoietic cells, non-hematopoietic cells, and non-cellular components is essential for the development of specialized niches which actively contribute to MM growth and to the development of drug resistance [172]. To date, efforts to expand primary MM cells outside their BM microenvironment have largely failed [173], confirming the importance of the BM niche in supporting MM proliferation and survival. 

Given the importance of the TME, the reported MM models often include non-cellular components of the tissue ECM, such as ECM proteins, either as coating on polymer scaffolds or as components of hydrogels in which cells are embedded. Most models also strive to replicate the interactions between MM cells and MSCs, either by simple co-culture or by exploiting more complex culture conditions, such as 3D structures and dynamic systems [167,170,174,175,176,177,178,179].

Trujillo et al. [180] developed an innovative 3D platform based on semi-solid culture to model the MM microenvironment. The model is a dynamic suspension of polymer microspheres coated with hyaluronic acid (HA) or fibronectin, two non-cellular components of the BM ECM [181,182], implicated in MM cells adhesion and in their consequent evolution into drug resistant cells [183]. The microspheres were incubated with three different MM non-adherent cells, namely RPMI8226, U226, and MM1.S, on spinning plates to create a dynamic environment compatible with the suspension-culture conditions required for MM cells. The cells, cultured in this dynamic environment or in traditional suspension culture, were treated with Dexamethasone or with Bortezomib, two common chemotherapeutic agents in MM [184,185]. The authors found an up to 20-fold increase in the proliferation rate of cells after treatment with Bortezomib, indicative of drug resistance, for the cells cultured in the dynamic model. On the contrary, no indications of drug resistance were observed in suspension cells, suggesting that a dynamic environment including ECM proteins may better replicate the interactions between MM cells and the TME which bring to the development of drug-refractory phenotypes.

Ferrarini et al. [186] used a Rotary Cell Culture System (RCCS™) Bioreactor to culture MM specimens ex vivo. The authors were able to maintain MM explants for up to 14 days within the bioreactor, reporting the presence of the different components of MM TME, such as MM cells and arteriolae, and preservation of the native tissue architecture. The authors also found that patient samples in the RCCS bioreactor maintained the original sensitivity to proteasome inhibition treatment, confirming the applicability of the model in drug evaluation and screening. 

Spelat et al. [187] used a different approach to maintain MM cells in co-culture with MSCs. In detail, they embedded MSCs into a thermo-responsive polymer hydrogel of poly(glycerol monomethacrylate)-block-poly-(2-hydroxypropyl methacrylate). Using a trans-well insert, the hydrogel loaded with MSCs was placed in a cell culture plate containing MM cells in suspension. Interestingly, the authors observed a significant over-expression of the chemokine receptor CX3CR1, which is known to mediate the interactions between MM and BM cells that favor cell survival and disease progression [188]. They also reported an upregulation of IL-6 and IL-10, which was correlated with an increment in MM cells proliferation, only in co-culture with MSCs. 

Recently, Wu et al. [171] proposed coaxial extrusion 3D bioprinting, to develop cannular constructs mimicking the BM cavity. The construct presented a hollow structure surrounded by a shell, mimicking the cortical bone, while MM cells (MM1S cell line) were printed in the core, alone or in combination with HS5 stromal cells, using a bioink composed of a blend of gelatin methacryloyl, alginate, polyethylene glycol-diacrylate, and nano-hydroxyapatite. Treatment response was analyzed by treating the 3D model with BTZ for 24 h. The authors reported significantly higher IC50 values of BTZ for MM cells in 3D co-culture with stromal cells, in comparison with 2D culture and with the 3D model hosting only MM cells, with a 3-fold and a 1.7-fold increase, respectively. They also observed that MM cells in the co-culture group showed aggregation following BTZ treatment. Co-administration of tocilizumab (TOC) to reduce cell adhesion was found to significantly improve BTZ treatment [189,190], highlighting the potential of this model as a screening for combinatorial therapeutic options. Moreover, the authors were able to maintain patient-derived MM cells for up to 7 days in the model, while survival in traditional 2D culture did not exceed 5 days [191]. 

### 3.3. Lymphoma In Vitro Models

As mentioned above, lymphomas include HL and NHL, which have different characteristic hallmarks. HL is characterized by the degradation of the lymph node structure caused by the presence of mutated B-cells, namely Hodgkin and Reed–Sternberg cells, which lead to defective immunoglobulin expression, and to the development of an inflammatory TME. HL cells constitute less than 1% of the total cell population while most cells are non-malignant reactive immune cells, which have been suggested to support HL progression [192]. 

NHLs arise from the genetic alteration of B-lymphocytes (86% of all NHL), T- cells, or NK-cells (14% of all NHL) [193]. Recent findings highlighted the key role of TME in NHL pathogenesis. Indeed, non-tumoral cells, such as immune cells, stromal cells, blood vessels, and extracellular components [194], together with cancer cells create a dynamic microenvironment that triggers tumor initiation, survival, proliferation, immune escape, and drug resistance [195,196,197,198]. For example, MSCs in the BM are known to interact with NHL cells, promoting an anti-inflammatory and immunosuppressive microenvironment that leads to cancer cell proliferation and to drug resistance [199,200].

Only a few models of HL and NHL have been developed to date, because of the difficulty of recreating such dynamic interplay among cells in the TME, as well as because of technical issues correlated with the poor survival of most cell types in vitro and to the co-culture conditions [201]. 

Bahlmann et al. [202] developed an in vitro model of HL that included TAMs, a fundamental immune component of the TME. To mimic the composition of the HL ECM, the authors developed a biomimetic cryogel composed of hyaluronan, gelatin, and fibronectin conjugated with adhesive peptides to facilitate TAMs invasion. The tendency of TAMs to invade the cryogel was investigated in co-culture with Reed–Sternberg cells or in culture with their conditioned medium. The authors observed that macrophages were able to invade the hydrogel to a significantly higher extent in co-culture with HL cells or when maintained in HL-conditioned medium, as compared to single TAMs culture in non-conditioned medium. TAMs were also cultured with conditioned medium from six different NHL cell lines, with the observation of a significantly lower gel colonization in comparison to macrophages cultured in HL cells-conditioned medium, confirming the different behavior of these two lymphoma sub-types. TAMs cultured in an HL cells-conditioned medium were treated with 25 therapeutic agents that interfere with HL-TAM communication and their gel colonization behaviour was monitored. The authors were able to identify five compounds that led to a significant reduction in macrophage colonization, namely the MMPs inhibitors Marimastat and Batimastat, the STAT 6 inhibitor (AS1517499), the P38-MAPK inhibitor (PD-169316), and the JAK1/2 inhibitor Ruxolitinib. The results showed that PD-169316 was most effective in inducing re-polarization of macrophages into a pro-inflammatory phenotype, while AS1517499 and Ruxolitinib significantly reduced the activity of the pro-inflammatory gene TNFA and of the M2-like gene CD206, respectively. As expected, PD-169316 caused a significant reduction of MMPs activity. The HL model, albeit including only two cell types of the TME, enabled the screening of a library of compounds and the identification of their key mechanisms of action, paving the way for the development of more efficient treatments.

Immune system-tumor crosstalk also plays a key role in NHL pathogenesis. Mannino et al. [203] developed an NHL model based on a HA hydrogel containing cancer and immune cells, integrated in a perfusable polydimethylsiloxane (PDMS) construct, which reproduced an endothelialized microchannel to mimic the tumor microvasculature. The channel was coated with mouse lung microvascular endothelial cells under rotation to achieve uniform cell attachment to the inner surface of the channel. VE-cadherin staining confirmed the full endothelialization of the channel. Administration of a model fluorescent antibody via direct injection in the microchannel resulted in antibody extravasation and permeation through the gel. Extravasation was significantly enhanced in the presence of NHL cells, confirming that vessel permeability is affected by cancer cells [204]. The therapeutic anti-CSF-1R antibody, which specifically targets macrophages [205], was also injected in the microchannel, resulting in a significant reduction of macrophage viability (of nearly 50%) in comparison to the untreated control sample. 

Ceccato et al. [206] proposed a model of NHL, using human decellularized femoral bone to replicate the BM microenvironment. In detail, decellularized scaffolds were seeded with BM stromal cells (HS-5) using a custom-made PDMS dual-step seeding device that allowed the slow-flowing of the cell suspension within the matrix. They found that NHL cells colonized the decellularized scaffold creating a strong interaction with MSCs and maintaining the expression of specific surface markers (e.g., CD19, CD20, and CD45). 

Treatment with Doxorubicin, clinically used for the treatment of DLBC, indicated a significantly lower cytotoxicity of the drug when administered in the model, as compared to drug treatment in traditional 2D cell culture. For instance, Doxorubicin-induced apoptosis on germinal B-cell-derived OCI-LY18 cells decreased from 49.9% + 7.7% in 2D culture to 30.7% + 9.2% in BM ECM model. When HS-5 were added in the BM ECM model, the apoptosis rate of NHL cells further decreased to 27.6% + 7.3%, confirming the importance of the TME in determining drug response. 

Recently Mastini et al. [207] used a commercial microfluidic chip (DAX-1, AIM Biotech) to develop a model of the perivascular niche in large cells anaplastic lymphoma. The microfluidic device was composed by two lateral channels, mimicking the blood vessels and a central channel, reproducing the perivascular niche. Endothelial cells (HUVECs) were seeded within the lateral channels with lymphoma cells flowing within the channel. 

The authors reported a reduced cytotoxic effect of the tyrosine kinase inhibitor crizotinib on large cells anaplastic lymphoma, when the cells were co-cultured with HUVECs, and they showed that this effect was mediated through the CCL19-21/CCR7 axis. This work confirmed the critical role of the TME in supporting the persistence of large cells anaplastic lymphoma cells after chemotherapy. 

## 4. Conclusions

In hematological malignancies, TME plays a fundamental role in orchestrating tumor progression and treatment response [208]. Therefore, TME should be considered an active player in the pathogenesis of BCs and an important therapeutic target. Unfortunately, its complex and heterogeneous composition, characterized by the presence of tumor-associated macrophages, endothelial cells, immune infiltrate, and other non-tumoral cell types, as well as of an altered ECM and modified cytokines expression, is hard to replicate in vitro [209].

Additional complexities include co-culturing issues associated with medium selection, short in vitro survival of some cell types, and non-attachment growth conditions, which are further exacerbated by the differences among BC subtypes.

For these reasons, few attempts have been made to generate complex in vitro 3D models of BCs. In this context, this review aims at encouraging engineering and exploitation of these largely unexplored systems, providing an updated overview of the state-of-the-art in vitro models of BCs, highlighting the main features of the developed models and identifying technological challenges and knowledge gaps in the field.

The resulting analysis of the literature shows that in vitro 3D models of BCs can replicate to some extent the interactions between different BC cells, endothelial cells, immune infiltrate, and stromal cells in the TME, which orchestrate tumor development and treatment response. These models have demonstrated the potential to extend the survival of short-living cells, to deepen the understanding of pathology development, and to offer reliable drug screening conditions, making them a valid alternative to 2D or 3D BC cells monocultures. The development of BC models may introduce alternative systems for those subtypes that still lack reliable and easy-to-establish animal models, such as tumors arising from central nervous system (CNS) infiltration of BC cells. 

Future research should focus on optimizing ECM modeling by better replicating its structure and protein composition, on integrating a functional vascular network, and on including additional cell types to better reflect the complexity of TME. Despite the evident need for further improvements, 3D models represent essential tools for the study of BC, with the ability to unravel the role of TME and to support the development of patient-specific therapies, demonstrating their incontrovertible potential. 

## Figures and Tables

**Table 1:** Therapeutic agents currently involved in BCs treatment.

Malignancy Subtype	Therapy	Therapeutic Agent
ALL	Chemotherapy	Vincristine, liposomal Vincristine, Daunorubicin, Doxorubicin, Cytarabine, L-asparaginase, PEG-L-asparaginase, 6- mercaptopurine (6-MP), Methotrexate, Cyclophosphamide, Prednisone, Dexamethasone, Nelarabine [38]
	Targeted therapy	Imatinib, Dasatinib, Nilotinib, Ponatinib, Bosutinib [39]
	Immunotherapy	Anti-CD20 Ab (Rituximab, Ofatumumab), Anti-CD22 Ab (Inotuzumab ozogamicin), Anti-CD19 Ab (Blinatumomab) [40,41,42,43,44,45]
	CAR-T cells	Anti-CD19 CAR-T cells Anti-CD19/CD22 CAR-T cells [46,47,48,49,50]
CLL	Chemotherapy	Fludarabine, Pentostatin, Cladribine Chlorambucil, Bendamustine Cyclophosphamide, Prednisone, Methylprednisolone, Dexamethasone [51]
	Targeted therapy	Ibrutinib, Acalabrutinib, Zanubrutinib, Pirtobrutinib, Idelalisib, Duvelisib, Venetoclax [52]
	Immunotherapy	Anti-CD54 Ab (Alemtuzumab) Anti-CD20 Ab (Obinutuzumab, Ofatumumab, Rituximab) [53]
	CAR-T cells	Anti-CD19 CAR-T cells, Anti-CD19/Anti-CD20 CAR-T cells [54,55]
AML	Chemotherapy	Cytarabine, Daunorubicin, Idarubicin, Cladribine, Fludarabine, Mitoxantrone, Etoposide, Hydroxyurea, Prednisone, Dexamethasone, Methotrexate, 6-mercaptopurine, Azacitidine, Decitabine, Liposomal daunorubicin, Cytarabine [56]
	Targeted therapy	Midostaurin, Quizartinib, Gilteritinib, Ivosidenib, Olutasidenib, Enasidenib, Venetoclax, Glasdegib [57]
	Immunotherapy	Anti-CD33 Ab (Gemtuzumab ozogamicin) [58]
	CAR-T cells	Anti-CD33 CAR-T-cell [59]
CML	Chemotherapy	Hydroxyurea, Methotrexate, Thioguanine, Chlorambucil, Cisplatin, Cyclophosphamide, Mechlorethamine, Etoposide [60]
	Targeted therapy	Imatinib, Dasatinib, Nilotinib, Bosutinib, Ponatinib, Asciminib [61]
	Immunotherapy	Anti-CD20 Ab (Rituximab) [62,63]
	CAR-T cells	Anti- IL-1RAP CAR T-cells [64]
MM	Chemotherapy	Cyclophosphamide, Etoposide, Doxorubicin, Liposomal doxorubicin, Melphalan, Bendamustine [65] Pomalidomide [66]
	Targeted therapy	Lenalidomide, Carfilzomib, Bortezomib [65]
	Immunotherapy	Anti-CD38 (Daratumumab, Isatuximab) Anti-SLAMF7 Ab (Elotuzumab) [65] Anti-BCMA (Elranatamab, Teclistamab) [67] Anti-GPRC5D (Talquetamab) [68]
	CAR-T cells	Anti-BCMA CAR-T-cell Anti-CD19 CAR-T-cell [69,70]
HL	Chemotherapy	Bleomycin, Doxorubicin, Cytarabine, Cyclophosphamide, Dacarbazine, Mechlorethamine, Procarbazine, Prednisone, Etoposide, Vinblastine, Vincristine [71]
	Immunotherapy	Anti-CD30 Ab (Brentuximab vedotin) Anti-CD20 Ab (Rituximab) [72] Anti-PD-1 (Nivolumab) [73]
	CAR-T cells	Anti-CD30 CAR-T-cell [74,75]
nHL	Chemotherapy	Cyclophosphamide, Chlorambucil, Bendamustine, Ifosfamide, Prednisone, Dexamethasone, Cisplatin, Carboplatin, Oxaliplatin, Fludarabine, Pentostatin, Cladribine, Cytarabine, Gemcitabine, Methotrexate, Pralatrexate,, Liposomal doxorubicin, Vincristine, Mitoxantrone, Etoposide (VP-16), Bleomycin [76]
	Targeted therapy	Idelalisib, Bortezomib, Belinostat, Ibrutinib, Acalabrutinib, Zanubrutinib, Pirtobrutinib, Duvelisib, Tazemetostat, Selinexor [77]
	Immunotherapy	Anti-CD30 Ab (Brentuximab vedotin), Anti-CD20 Ab (Obinutuzumab, Rituximab, Ofatumumab), Anti-CD79b Ab (Polatuzumab vedotin) Anti-CD52 Ab (Alemtuzumab), Anti-CD19 Ab (Loncastuximab tesirine) Bispecific Ab (Mosunetuzumab, Epcoritamab, Glofitamab) [78]
	CAR-T cells	Anti-CD19 CAR-T-cell [78]

## Data Availability

No new data were created or analyzed in this study. Data sharing is not applicable to this article.
